# The role of mitochondrial function in gold nanoparticle mediated radiosensitisation

**DOI:** 10.1186/s12645-014-0005-7

**Published:** 2014-09-16

**Authors:** Laura E Taggart, Stephen J McMahon, Fred J Currell, Kevin M Prise, Karl T Butterworth

**Affiliations:** Centre for Cancer Research and Cell Biology, Queen’s University Belfast, Queen’s, BT9 7AE Northern Ireland; School of Mathematics and Physics, Queen’s University Belfast, Queen’s, BT7 1NN Northern Ireland

**Keywords:** Gold nanoparticles, Radiosensitisation, Radiation, Mitochondria, Oxidative stress

## Abstract

Gold nanoparticles (GNPs), have been demonstrated as effective preclinical radiosensitising agents in a range of cell models and radiation sources. These studies have also highlighted difficulty in predicted cellular radiobiological responses mediated by GNPs, based on physical assumptions alone, and therefore suggest a significant underlying biological component of response. This study aimed to determine the role of mitochondrial function in GNP radiosensitisation. Using assays of DNA damage and mitochondrial function through levels of oxidation and loss of membrane potential, we demonstrate a potential role of mitochondria as a central biological mechanism of GNP mediated radiosensitisation.

## Background

The application of radiobiological principles in clinical oncology aims to describe the relationship between absorbed dose and the resulting biological responses of tumour and normal tissues (Hall & Giaccia 2012). Central to the development of novel clinic approaches is improvement in the differential responses between normal and tumour tissue at a fixed dose, termed the therapeutic ratio. Improvements in the therapeutic ratio of radiotherapy have been driven by developments in both radiation biology and radiation physics which have translated into significant advances in targeted dose delivery, radiological imaging and biological effectiveness.

Since the pioneering attempts of Denekamp and colleagues in the mid-1970s to sensitize hypoxic tumour cells (Fowler et al. 1976), much effort has focussed on increasing tumour cell sensitivity to the biological effects of ionising radiation (Wardman 2007). In the nanotechnology field, gold nanoparticles (GNPs) have been extensively investigated as radiosensitisers, reviewed by our laboratory (Butterworth et al. 2012); and have recently shown efficacy under hypoxic conditions (Jain et al. 2014). GNPs are applicable as radiosensitsers due to their high atomic number (Z = 79) which results in preferential mass energy absorption compared to soft tissue (Hubbell & Seltzer 1996). Additionally, GNPs are relatively easy to synthesize in a range of sizes, can be readily functionalised, and have been shown to passively accumulate in tumours through the enhance permeability and retention effect (EPR) (Maeda et al. 2000).

Calculations of X-ray dose enhancement factors based on physical absorption characteristics have predicted enhancements of between 1.2 and 5 depending on the GNP concentration and beam energy, with the greatest effect predicted at kilovoltage energies (Cho 2005; McMahon et al. 2008). Despite these predictions radiosensitisation of cells exposed to GNPs and irradiated with megavoltage energies has been shown suggesting additional processes in the radiosensitising effect of GNPs (Chithrani et al. 2010; Jain et al. 2011). In addition to possible biological mechanisms, one factor which may contribute to these effects is localised energy deposition around GNPs. Following ionisation of gold atoms, large numbers of low-energy electrons are generated through Auger cascades which deposit their energy at high density within a small radius around the GNP, leading to high localised doses. These high, inhomogeneous doses generated in close proximity to the nanoparticle surface are known to have significantly increased biological effectiveness with analysis of nanoscale dose distributions around GNPs using the Local Effect Model (McMahon et al. 2011a; McMahon et al. 2011b) suggesting this may contribute to the observed radiosensitising effects of GNPs.

Of the wide ranging studies describing the biological effects of GNPs, several have reported elevated levels of reactive oxygen species for GNPs of differing size, shape and surface functionalization (Pan et al. 2009; Chompoosor et al. 2010; Li et al. 2010; Piryazev et al. 2013; Mateo et al. 2014). Comparatively few reports have demonstrated a role for ROS or the involvement of mitochondria as mechanism of GNP radiosensitisation (Geng et al. 2011). The current study builds on previous data from our laboratory demonstrating radiosensitising effects of 1.9 nm Aurovist GNPs at kilovoltage energies (Butterworth et al. 2010) as a result of significantly elevated levels of DNA damage which may be a direct result of impaired mitochondrial functional manifested by increased oxidation and loss of membrane potential.

## Materials and methods

### Cell culture

All cell lines were obtained from Cancer Research UK. The human breast cancer cell line, MDA-MB-231 was maintained in Dulbecco’s modified Eagle’s medium (DMEM) supplemented with 10% foetal bovine serum and 50 μg/ml penicillin/streptomycin. The human prostate cell line, DU-145 was maintained in RPMI-1640 medium with 10% foetal bovine serum and 50 μg/ml penicillin/streptomycin. The human glioma cell line, T98G was maintained in EMEM supplemented with 10% foetal bovine serum and 50 μg/ml penicillin/streptomycin.

### Gold nanoparticles

1.9 nm Aurovist^TM^ particles were purchased from Nanoprobes Inc. (NY, USA) and re-suspended in sterile water. 1.9 nm Aurovist^TM^ are spherical particles with a proprietary thiol coating (Coulter et al. 2012). Cells were treated at a concentration of 500 μg/ml for 24 hours unless otherwise indicated. This concentration of 500 μg/ml and time point of 24 hours was chosen as a result of previous work within the group showing that these conditions allow for optimal cell uptake of GNPs (Coulter et al. 2012).

### Cell Irradiation

Cells were irradiated with 225 kVp X-rays produced using an X-Rad 225 X-ray generator (Precision, X-ray Inc, USA). All quoted doses are the absorbed dose from this source in water.

### Clonogenic cell survival assay

Sub-confluent cells were removed from flasks using a solution of 0.25% Trypsin and 1 mM EDTA, they were counted using a Coulter counter and re-seeded into six well plates at a density of 1.5 x 10^5^ cells per well. Cells were left to attach for 4–6 hours and treated with gold nanoparticles for 24 hours. Cells were then irradiated, trypsinised and counted, then seeded into T25 flasks and left to proliferate for 7–9 days. For MDA-MB-231, DU145 and T98G cell lines 500 cells were seeded per treatment for 0 Gy and 2 Gy doses, 1,000 cells for 4 Gy and 2,000 cells for 8 Gy. MDA-MB-231, DU-145 and T98G cells had plating efficiencies of approximately 50%. Surviving fraction was calculated by dividing the number of surviving colonies in the irradiated samples by the number of surviving colonies in the non-irradiated controls for each treatment. Dose enhancement factor (DEF) is defined here as the ratio of doses which lead to equal levels of cell survival with and without GNPs. DEFs can vary with delivered dose, and are quoted with reference to the dose delivered to cells in the absence of GNPs.

### Immunofluorescent microscopy

Cells were seeded onto sterile 16 mm^2^ coverslips placed in six well plates at a density of 1 x 10^5^ cells per well. Cells were left to attach for 4–6 hours before treatment. After incubation with GNPs cells were irradiated with 2 Gy and fixed 1 hour or 24 hours post irradiation with a 50% acetone/50% methanol solution for 10 minutes. Cells were then permeabilised with 0.5% Triton X-100 and PBS solution for 10 minutes before being incubated with a blocking buffer of 0.2% milk, 5% Horse serum, 0.1% Triton X-100 in PBS for 1 hour at room temperature. Coverslips were then incubated with 53BP1 antibody (Novus Biologicals, Colorado, USA) at a dilution of 1:1000 in blocking buffer for 1 hour at room temperature. They were then rinsed three times with washing buffer, 0.1% Triton X-100 in PBS before being incubated with Alexa Fluor 488 Goat anti Rabbit secondary antibody (Invitrogen Molecular Probes, Oregon, USA) at a dilution of 1:1000 in blocking buffer for one hour at room temperature. Coverslips were rinsed three times in washing buffer and then mounted onto glass microscope slides with 5 μl of Vectashield mounting media (Vector Labs Ltd, UK) and sealed with nail varnish. Foci were viewed and counted manually on a Zeiss Axiovert 200 M fluorescent microscope.

### Mitochondrial membrane polarisation measurement

Cells were seeded into 12 well plates at a density of 1 × 10^5^ cells per well and left to attach for 4–6 hours before treatment. 25 nM Tetramethylrhodamine ethyl ester perchlorate (TMRE) (Sigma-Aldrich) was added to each well and incubated for 15 minutes at 37°C. Media was then transferred to 15 ml centrifuge tubes and placed on ice. Cells were detached using 0.25% trypsin and 1 mM EDTA and the cell solution was then transferred to the corresponding 15 ml tube left on ice. Cells were then pelleted by centrifugation at 2000 rpm at 4°C for 5 minutes. Media was removed and cell pellets were resuspended in 300 μl of PBS and TMRE fluorescence was analysed immediately using a FACSCalibur flow cytometer with an air-cooled argon-ion 15 milliwat 488 nm laser and 585 nm detector and CELL-Quest software (BD biosciences) 1 x 10^4^ cells were analysed per sample.

### Mitochondrial oxidation detection

Mitochondrial oxidation was measured using Nonyl-Acridine Orange (NAO) (cat no A-1372, Molecular Probes, Invitrogen, NY). 1 × 10^5^ cells were seeded into 12 well plates and left to attach for 4–6 hours before being treated accordingly. At the end of treatment, media was removed from cells and transferred to 15 ml centrifuge tubes on ice. Cells were detached using 0.25% Tryspin/1 mM EDTA solution and added to corresponding tubes containing media. Cells were then pelleted by centrifugation at 2000 rpm at 4°C for 5 minutes. Media was removed and cell pellets were resuspended in 300 μl of 0.1% BSA-PBS solution containing 25 ng/ml NAO and left to incubate at 37° for 10 minutes. Cells were placed on ice post-incubation and analysed immediately using FACSCalibur flow cytometer with an air-cooled argon-ion 15 milliwatt 488 nm laser and 585 nm detector and CELL-Quest software (BD biosciences). 1 × 10^4^ cells were analysed per sample.

## Results

### Radiosensitising effects of 1.9 nm GNPs

**Figure 1:**
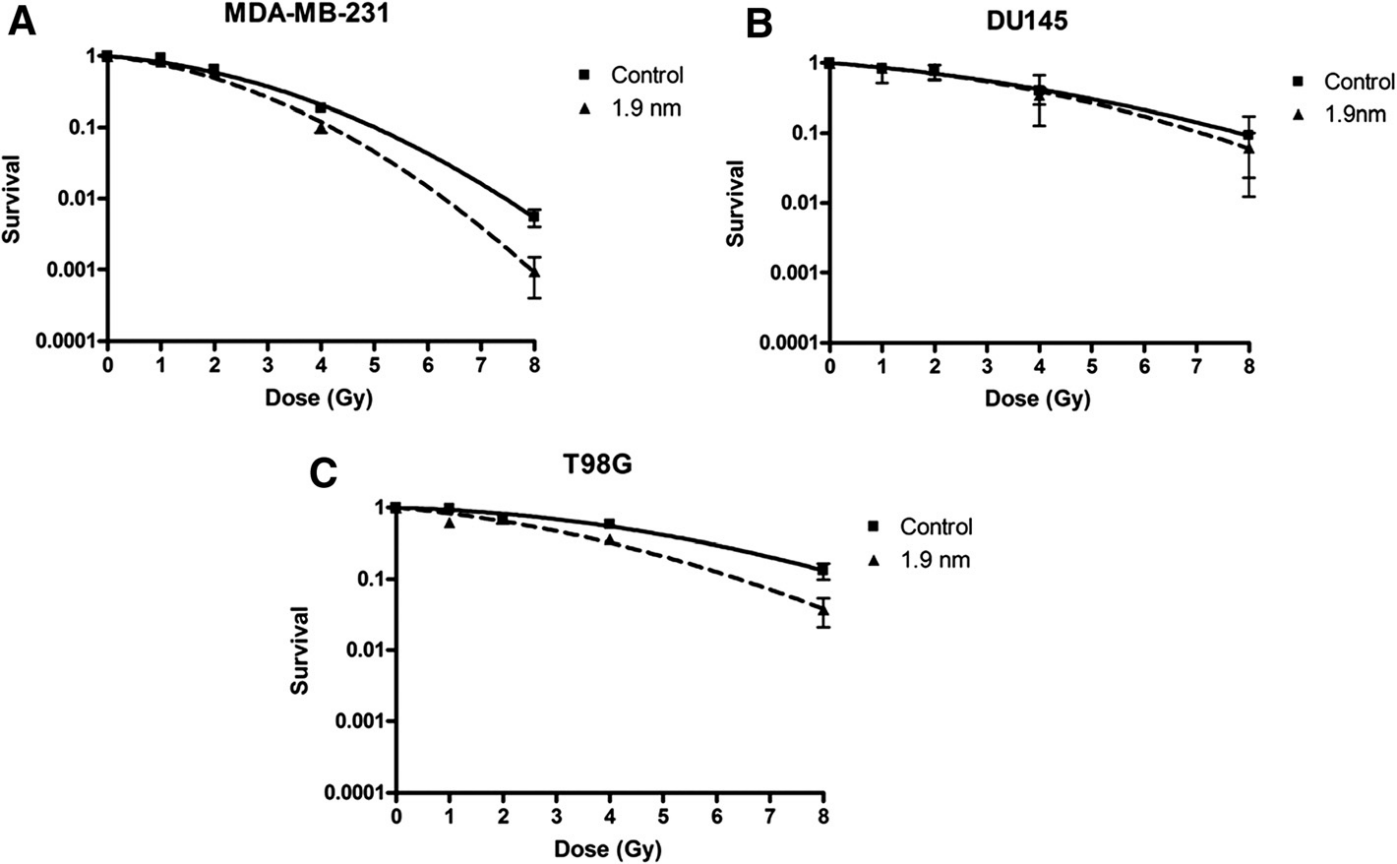
**Radiation dose response curves for (A) MDA-MB-231, (B) DU145 and (C) T98G cells treated with 500 μg/ml of 1.9 nm gold nanoparticles (GNPs) 24 hours prior to irradiation with 225 kVp x-rays.** Experiments were performed at least three times in triplicate, means are presented ± standard error of the mean.

To assess the efficacy of 1.9 nm GNPs as radiosensitisers, clonogenic cell survival assays were performed in three cancer cell lines. Cells were treated with 500 μg/ml of Aurovist^TM^ added to the culture medium 24 hours prior to irradiation with 225 kVp X-rays (Figure 1). GNP concentrations and incubation time were chosen to complement previous studies from our laboratory (Jain et al. 2011; Coulter et al. 2012). Dose enhancement factors (DEFs) were calculated as the ratio of doses leading to equal levels of cell survival in the presence and absence of GNPs. DEFs can vary with delivered dose and are quoted with reference to the dose delivered to cells in the absence of GNPs. Table 1 summarizes the DEF for each cell line and gold nanoparticle preparation for 2 Gy, 4 Gy and 8 Gy doses.

**Table 1:** **Summary of dose enhancement factors (DEF) ± uncertainties for the cell lines investigated when irradiated at 2 Gy, 4 Gy and 8 Gy after treatment with 1.9 nm gold nanoparticles**

	**MDA-MB-231**	**DU145**	**T98G**
2 Gy	1.23 ± 0.14	1.01 ± 0.20	1.90 ± 0.22
4 Gy	1.20 ± 0.06	1.06 ± 0.10	1.57 ± 0.08
8 Gy	1.17 ± 0.02	1.1 ± 0.04	1.35 ± 0.03

DEF is defined here as the ratio of doses which lead to equal levels of cell survival with and without GNPs. DEFs can vary with delivered dose, and are quoted with reference to the dose delivered to cells in the absence of GNPs.

Significant radiosensitising effects were observed in both MDA-MB-231 and T98G cell lines with 1.9 nm GNPs but not DU-145 cells as shown in Figure 1. T98G glioma cells show the greatest amount of cell death enhancement with a DEF of 1.90 ± 0.22 at 2 Gy with 1.9 nm GNPs. MDA-MB-231 cells also show increased cell kill with GNPs with a lower DEF of 1.23 ± 0.14 at 2 Gy compared to T98G cells. DU-145 cells show virtually no change in cell survival across all doses investigated. It should also be noted that in the T98G cell line, GNP DEFs appear to decrease with increasing dose; at 8 Gy the DEF decreased to 1.35 ± 0.03, suggesting GNPs are not solely acting as a dose modifying agent as DEFs would be expected to be uniform across all doses in this case.

### GNP induced changes in DNA damage

**Figure 2:**
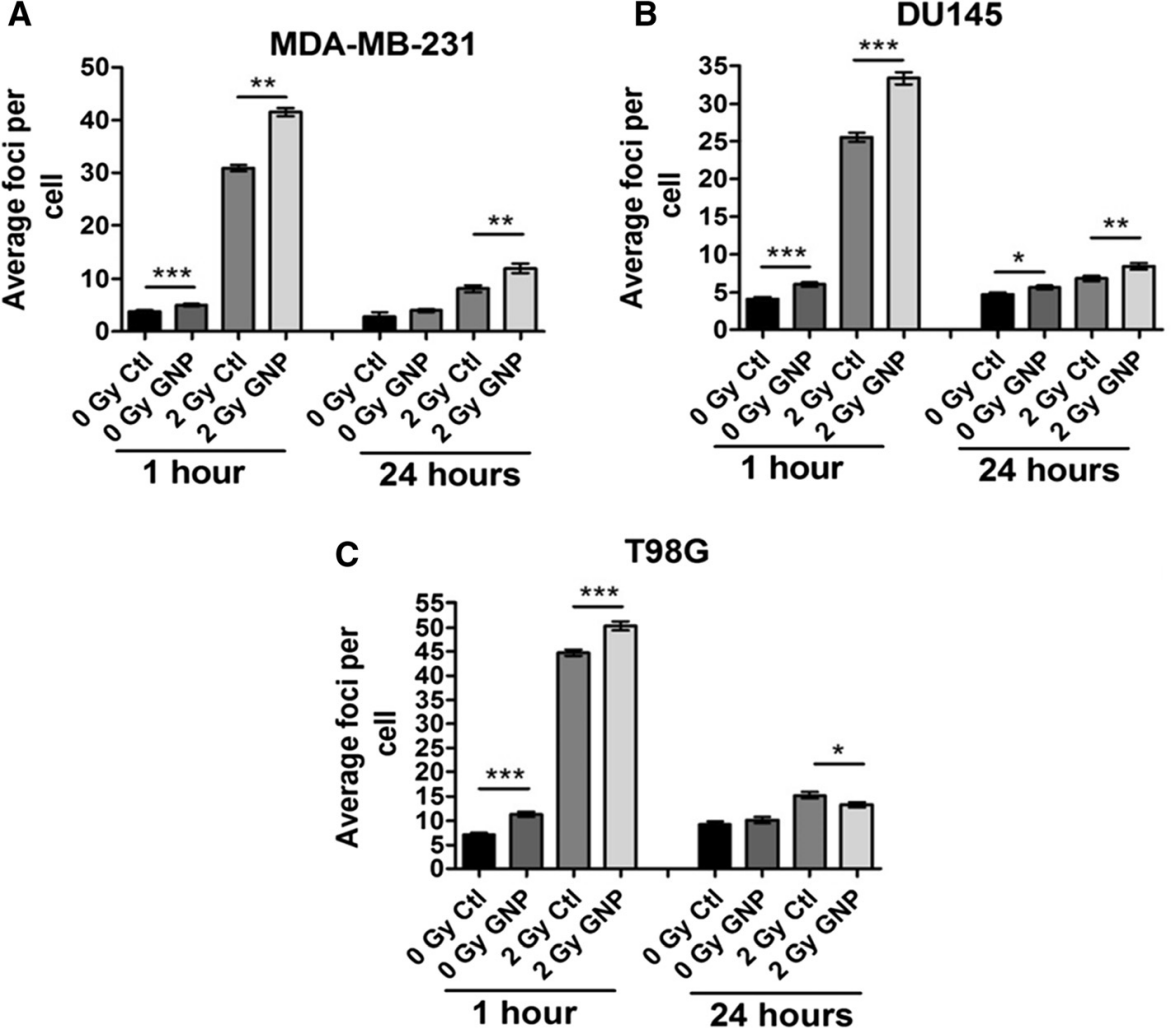
**DNA damage analysis measured by immunofluorescent staining of 53BP1 foci for (A) MDA-MB-231, (B) DU145 and (C) T98G cells treated with 500 μg/ml of 1.9 nm gold nanoparticles (GNPs), 24 hours prior to irradiation with 225 kVp X-rays at a dose of 2 Gy.** Cells were fixed, stained and foci scored at 1 hour and 24 hours post irradiation. For each of the experimental conditions, foci were scored in > 50 nuclei. Experiments were performed at least three times in triplicate, means are presented ± standard error of the mean. Statistical analysis was performed using a paired t-test with significant differences assumed at the level of *p = ≤ 0.05.

DNA damage was assessed by imaging and counting immunofluorescently stained 53BP1 foci in cells seeded onto glass coverslips. 53BP1 binds to tumour suppressor protein p53 and has been shown to accumulate at the sites of DNA damage and is required for the initiation of DNA repair (Wang et al. 2002). Figure 2 shows levels of DNA damage in MDA-MB-231, DU145 and T98G cells 1 hour and 24 hours after irradiation with and without GNPs. As shown in Figure 2, DNA damage increases following exposure to GNPs in the absence of radiation across all cell lines with increases of 30, 45 and 39% observed in MDA-MB-231, DU145 and T98G cells respectively. Increased levels of DNA damage were also present at 1 and 24 hours post irradiation with 2 Gy in MDA-MB-231 and DU-145 cells, but not T98G cells which showed a significant decrease in DNA damage in the presence of GNPs at 24 hours post irradiation. The residual DNA damage present 24 hours post irradiation with GNPs in MDA-MB-231 and DU145 cells suggests complex damage which hasn’t been repaired or an inability to repair the damage.

**Figure 3:**
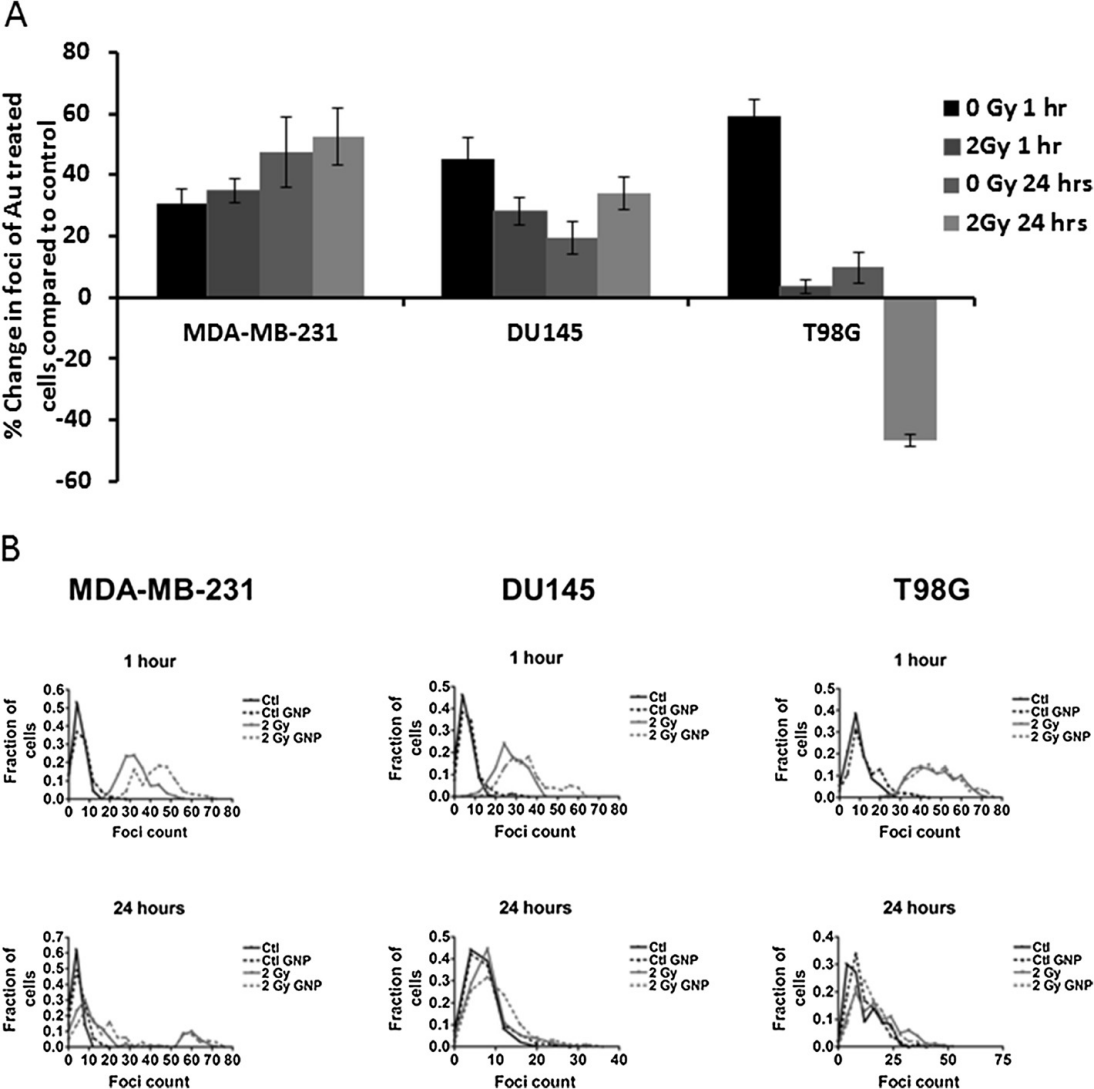
**Percentage change in DNA damage and distribution of damage foci per cells treated with 500 μg/ml of 1.9 nm gold nanoparticles (GNPs) 24 hours prior to irradiation with 225 kVp x-rays at a dose of 2 Gy.** Cells were fixed, stained and foci scored at 1 hour and 24 hours post irradiation. **(A)** Percentage change in average foci per cell for GNP exposed cells compared to control cells of the same condition calculated for both irradiated and non-irradiated cells. **(B)** Distributions of 53BP1 foci in cells (i) MDA-MB-231, (ii) DU145 and (iii) T98G cells. All figures are representations of foci data presented in Figure 2.

To determine if radiation induced effects were additive to the DNA damage induced solely by GNPs the percentage variation in DNA damage induced by GNPs was compared to the percentage increase in DNA damage caused by GNPs and irradiation as in Figure 3A. In the absence of irradiation, GNP treatment results in a 30% enhancement of DNA damage foci in MDA-MB-231 cells compared to a 34% enhancement at 1 hour post irradiation suggesting the observed enhancement post irradiation is due to an extension of the original damage caused by GNP treatment and not an additive effect of radiation exposure.

Furthermore, the distribution of foci numbers per cell was analysed in Figure 3B in order to determine if there was an overall increase in the levels of DNA damage across the population or if a subset of the population with a significant increase in DNA damage was driving the increase in average foci number. MDA-MB-231 and DU-145 cells both show a slight shift in a population subset with a peak of increased DNA damage when cells are treated with GNPs, which is further amplified with irradiation. T98G cells also show a slight peak shift towards additional damage upon nanoparticle treatment, but not in the presence of radiation.

### GNP induced changes in mitochondrial membrane polarisation

**Figure 4:**
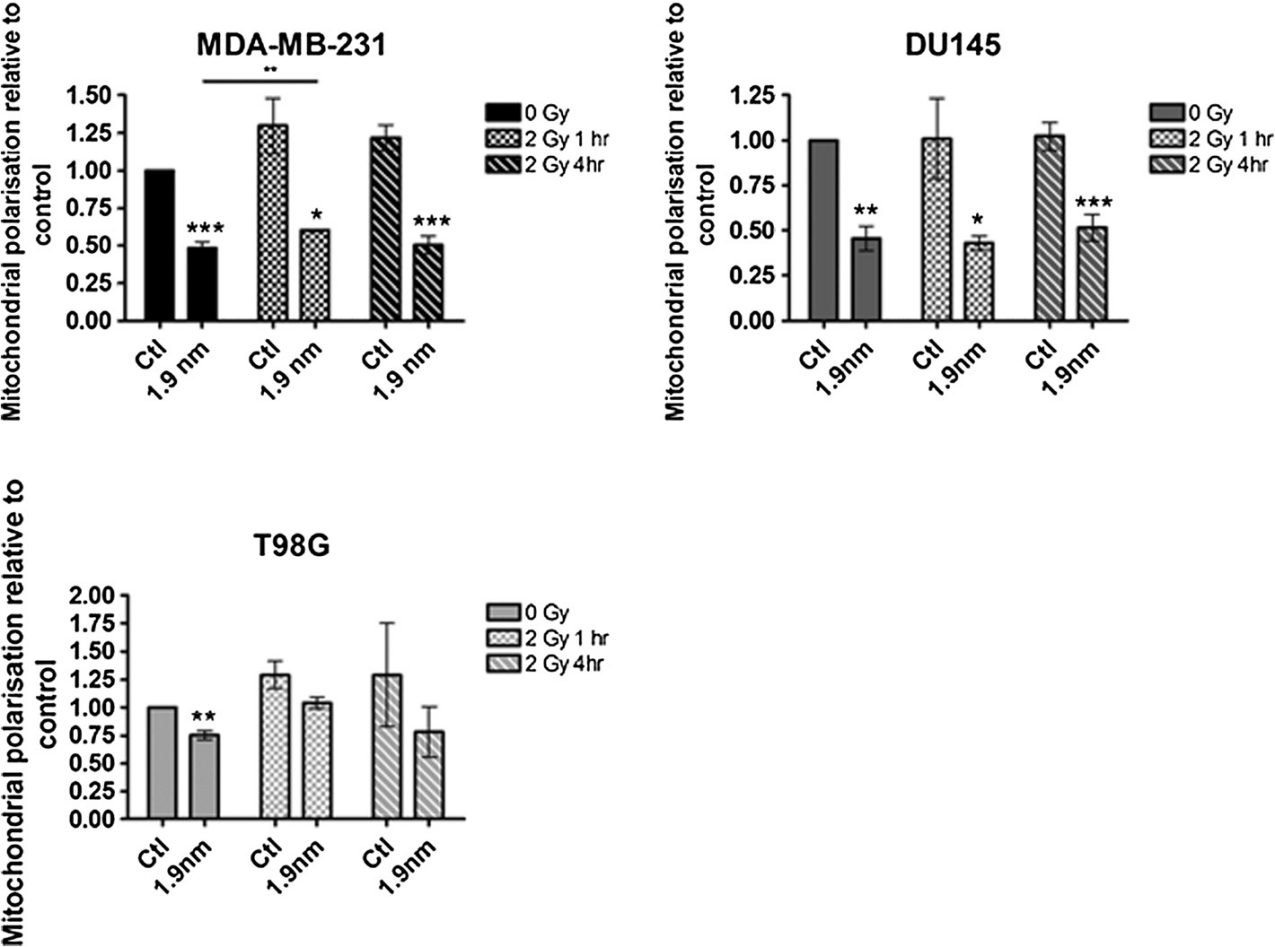
**Mitochondrial membrane polarisation after GNP and irradiation.** Mitochondrial membrane polarisation was measured by TMRE flow cytometry and made relative to untreated control after cells were treated with 1.9 nm GNPs and/or 2 Gy irradiation. Means are presented ± standard error of the mean. n = 5. Significance was measured by paired t tests against controls. A line between two bars with asterix denotes significant differences between two conditions. *p = ≤0.05, **p = ≤0.01, ***p = ≤0.001.

Changes in mitochondrial membrane polarisation were measured by flow cytometry analysis following 24 hours exposure to GNPs with and without exposure to a single dose of 2 Gy (Figure 4). In irradiated samples, depolarisation was measured 1 and 4 hours post irradiation. In unirradiated cells, GNPs alone significantly reduced mitochondrial membrane polarisation relative to controls across all cell lines with decreases of 50%, 55% and 25% in TMRE fluorescence in MDA-MB-231, DU-145 and T98G cells respectively. MDA-MB-231 and T98G cells both displayed an increase in mitochondrial membrane polarisation of 30% and 25% respectively, 1 hour post irradiation when exposed to GNP in combination with 2 Gy irradiation, which was significant in MDA-MB-231 cells, however, this coincided with an increase in membrane polarisation upon irradiation alone. At 4 hours post irradiation with GNPs membrane polarisation had returned to the same level as non-irradiated GNP treated samples across all cell lines.

### GNP induced changes in mitochondrial membrane oxidation

**Figure 5:**
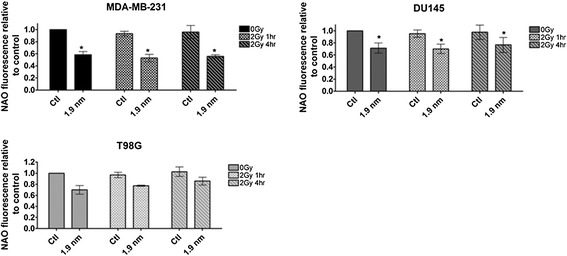
**Mitochondrial oxidation after GNP and irradiation.** Mitochondrial oxidation was measured by NAO flow cytometry and made relative to untreated control after cells were treated with 1.9 nm GNPs and/or 2 Gy irradiation. Means are presented ± standard error of the mean. n = 3. Significance was measured by paired t tests against controls. *p = ≤0.05.

Mitochondrial oxidation was measured by NAO fluorescent flow cytometry analysis 1 and 4 hours post 2 Gy irradiation following 24 hour exposure to GNPs (Figure 5). Similar reductions in fluorescence of NAO indicating mitochondrial oxidation were observed as seen previously with 40%, 45% and 25% reduction in fluorescence after GNP exposure in MDA-MB-231, DU145 and T98Gs respectively. These levels of oxidation remained consistent at both time points following irradiation indicating no significant change in mitochondrial oxidation.

## Discussion

Classical approaches used to radiosensitise cells have included radiation induced activation of prodrugs, suppression of intracellular thiols, inhibition of DNA repair and oxygen mimetics (Wardman 2007). Nitrobenzenes, nitrofurans and nitroimidazoles have been used to radiosensitise hypoxic cells with their radiosensiting ability attributed to their high electron affinity (Adams & Cooke 1969). These compounds are generally activated by reduction in hypoxic conditions and work in a similar way to oxygen by causing DNA double strand breaks in the presence of irradiation as a result of the fixation of free radical damage (Katz et al. 2009). Despite extensive preclinical research and promising evidence, hypoxic radiosensitisers have failed to reach their full potential in the clinic (Bischoff et al. 2009).

The concept of targeting repair DNA stems from the central dogma underpinning radiotherapy, which is to induce complex DNA damage lesions which are difficult to repair resulting in cell death. Cisplatin and 5-fluorouracil exemplify radiosensitisers in clinical use, acting by interfering with DNA synthesis, however, their precise mechanism of action in radiosensitisation is not fully understood (Katz et al. 2009).

Similarly, whilst GNPs have been demonstrated as effective radiosensitisers at a range of photon energies, there is insufficient explanation of their underlying biological mechanism of action (Butterworth et al. 2012). In this study we further validate previous reports from our laboratory showing significant radiosensitising effects of GNPs at 225 kVp (Butterworth et al. 2010). Analysis of DNA damage foci distributions from Figure 3B compared to foci scores in Figures 2 and 3A, shows the increased DNA damage following treatment with GNPs alone appears to be a result of a small shift in the observed levels of DNA damage within the whole cell population. In contrast, the increased levels of DNA damage seen after irradiation with GNPs appeared to be a result of a cell population subset with greatly amplified levels of DNA damage rather than the whole population. This is particularly obvious in MDA-MB-231 cells and can be seen at 1 and 24 hours post irradiation. This could be a result of the induction of oxidative stress which has previously been observed in our laboratory for the same GNPs (Butterworth et al. 2010).

To further determine the biological mechanism of GNP mediated radiosensitisation, this study considered the mitochondria as an extra-nuclear target for GNPs within the cell. Mitochondria have multiple roles in important cellular functions, including the production of adenosine triphosphate (ATP), cell signalling, cell growth, cell cycle progression and cell death (Raimundo 2014). In this study we clearly demonstrate GNPs to have a significant impact on mitochondrial function, manifested by oxidation of the mitochondrial membrane protein, cardiolipin and cell specific disruption of mitochondrial membrane potential. Although these effects could be driven by direct physical interaction with mitochondrial proteins and enzymes, this study supports an indirect interaction of GNPs with mitochondria, triggered by whole cell chemical processes such as oxidative stress. Additional experimental studies are required to further elucidate the precise mechanism of interaction.

Mitochondrial membrane depolarisation can be caused by the presence of free radicals, high intracellular calcium concentrations or stress of the endoplasmic reticulum (Gunter & Pfeiffer 2009; Deniaud et al. 2008). Considering the various reports of GNPs causing the induction of ROS and specifically the GNPs used in our experiments, it is likely that elevated ROS result in mitochondrial depolarisation (Butterworth et al. 2010). Mitochondria and mitochondrial function can be downstream targets of oxidative stress which impairs their function, and they themselves can produce reactive oxygen species and induce oxidative stress in the cell (Zorov et al. 2006). The effect of GNPs on mitochondrial processes could be a direct contributor to the DNA damage seen upon exposure to gold nanoparticles, as mitochondria have been shown to play a role in the induction of DNA damage (Tartier et al. 2007).

Oxidative stress and mitochondrial depolarisation are often significant cellular events preceding the induction of cell death, particularly by apoptosis. A key step in the initiation of the intrinsic apoptotic pathway is the oxidation of cardiolipin, which is assessed in this study by measuring the binding of the fluorescent compound NAO through flow cytometry. The oxidation of cardiolipin releases cytochrome c into the cytosol initiating apoptosis; this has been described as critical point in apoptotic signalling beyond which the cell is terminally committed to die (Jiang et al. 2008). Significant loss of fluorescence from nonyl-acridine orange in both MDA-MB-231 and T98G cells indicates oxidation of cardiolipin. Some loss of NAO fluorescence was also observed in DU-145 cells however, the level was not statistically significant.

**Figure 6:**
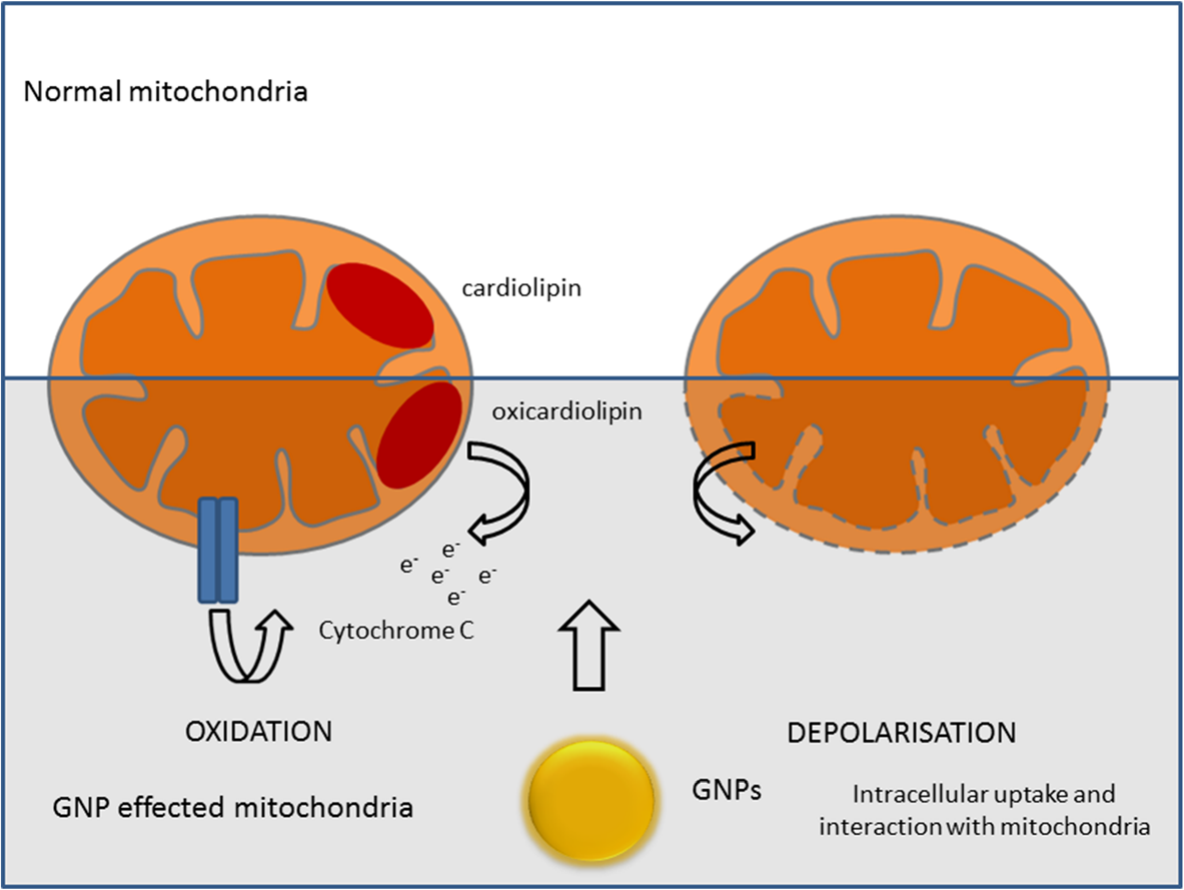
**Schematic representation of gold nanoparticle (GNP) radiosensitisation through mitochondrial function.**

In order to test the hypothesis that mitochondrial responses are initiated by GNPs and may predispose cells to radiosensitisation, it was important to confirm that these responses were not altered with the addition of irradiation. Figures 4 and 5 show no additional change in response to radiation in combination with GNP compared to GNPs alone, similar to the DNA damage data in Figure 2. Comparable to mitochondrial membrane polarisation, levels of cardiolipin oxidation remain steady post irradiation. However, the lack of change in the levels of mitochondrial membrane polarisation and cardiolipin post irradiation further emphasises the significance of the cellular events prior to irradiation in GNP radiosensitisation. As summarised in Figure 6, we propose the mitochondria as having a central role in biological response to GNPs alone and in combination with ionising radiation.

## Conclusions

1.9 nm gold nanoparticles are effective radiosensitisers showing significant decreases cell survival. In the absence of ionising radiation, GNPs have effects on DNA damage levels as well as mitochondrial function. These cell specific responses to GNPs have the potential to provide a biological mechanism for the sensitisation of cells to the effects of ionising radiation. This mitochondria mediated enhancement in cell death may in part explain the disparities between predicted physical dose enhancement and observed biological effect.

## Abbreviations

ATP: Adenosine triphosphate
DEF: Dose enhancement factor
DNA: Deoxyribonucleic acid
GNP: Gold nanoparticle
NAO: Nonyl-acridine orange
ROS: Reactive oxygen species
TMRE: Tetramethylrhodamine ethyl ester perchlorate
